# Immune modulation in gastric cancer: from macrophage polarization to immunotherapy

**DOI:** 10.3389/fimmu.2026.1711076

**Published:** 2026-08-11

**Authors:** Xiaofeng Cui, Xuerui Wang, Nan Jiang, Fangwei Zong, Weida Guan

**Affiliations:** 1 Department of Gastrointestinal Colorectal and Anal Surgery, China-Japan Union Hospital of Jilin University, Changchun, Jilin, China; 2 Department of Clinical Laboratory, The Second Hospital of Jilin University, Changchun, Jilin, China; 3 Department of Cardiovascular, The Second Hospital of Jilin University, Changchun, Jilin, China; 4 Department of Ophthalmology, The Second Hospital of Jilin University, Changchun, Jilin, China; 5 Department of Gastrointestinal surgery, Tonghua Central Hospital, Tonghua, Jilin, China

**Keywords:** gastric cancer, immune checkpoint inhibitors, immunotherapy, tumor microenvironment, tumor-associated macrophages

## Abstract

Gastric cancer remains a highly lethal malignancy characterized by late diagnosis, limited therapeutic responsiveness, and a profoundly immunosuppressive tumor microenvironment. Among the diverse cellular components shaping this ecosystem, tumor-associated macrophages (TAMs) have emerged as central orchestrators of gastric carcinogenesis, metastatic dissemination, and therapeutic resistance. TAMs promote tumor initiation through inflammatory recruitment and polarization, facilitate invasion and angiogenesis via cytokines, matrix-remodeling enzymes, and exosomal cargo, and impair antitumor immunity by suppressing T-cell and natural killer cell function. In addition, TAMs contribute to resistance to immune checkpoint blockade by sustaining an immunosuppressive tumor immune microenvironment enriched in Tregs, myeloid-derived suppressor cells, and inhibitory mediators such as TGF-β, IL-10, and PD-L1. Recent advances further highlight the translational promise of macrophage-targeted strategies, including polarization reprogramming, recruitment blockade, and chimeric antigen receptor macrophage therapy. Previous reviews primarily focus on the general biological roles of TAMs, this review synthesizes newly emerging mechanisms of TAM-mediated immunotherapy resistance, including spatial heterogeneity, stromal-vascular remodeling, and exosomal crosstalk, with the latest clinical advances in macrophage-directed immunotherapies. We specifically highlight the translational potential and current clinical trial landscape of chimeric antigen receptor macrophage (CAR-M) therapy and targeted reprogramming strategies, providing a forward-looking perspective on overcoming immune checkpoint blockade resistance in gastric cancer.

## 1 Introduction

Gastric cancer (GC) remains one of the leading causes of cancer-related mortality worldwide despite advances in surgery, chemotherapy, targeted therapy, and immune checkpoint inhibitors (ICIs) (1–3). Although immunotherapy has improved outcomes in selected patients, the overall clinical benefit remains limited because most GC exhibits an immunologically “cold” phenotype characterized by low T-cell infiltration, extensive stromal remodeling, suppressive cytokine signaling, and poor antigen presentation (4–6). These features establish a highly immunosuppressive tumor microenvironment (TME) that promotes immune evasion, therapeutic resistance, and disease progression. Among the diverse immune populations within the TME, tumor-associated macrophages (TAMs) are highly abundant and display remarkable functional plasticity (7, 8).

While classically activated M1 macrophages support antitumor immunity, M2-like TAMs promote tumor growth, angiogenesis, metastasis, and immune suppression through cytokine secretion, extracellular matrix remodeling, and crosstalk with T cells, fibroblasts, and endothelial cells (9, 10). Emerging evidence further demonstrates substantial spatial and functional heterogeneity of TAMs and highlights their central role in resistance to ICIs (11, 12). This review aims to delineate the multifactorial roles of TAMs in gastric carcinogenesis and uniquely bridges the gap between newly emerging mechanistic insights and recent clinical advances in macrophage-directed immunotherapies, with a specific focus on CAR-M therapy and strategies to overcome immunotherapy resistance.

## 2 TAM contributions to gastric carcinogenesis

### 2.1 Initial macrophage infiltration in gastric cancer

At the early stages of GC, tumor-secreted attractants such as osteopontin (OPN) and CCL2 initiate the recruitment of TAMs, while IL-33 stimulates mast cells to produce inflammatory mediators including IL-6, IL-13, and macrophage colony-stimulating factor (M-CSF), thereby intensifying monocyte migration into the tumor site (13, 14). The trajectory of macrophage differentiation is intricately tied to malignant progression. Helicobacter pylori infection upregulates COX-2/PGE2 and CCL2, which augments macrophage chemotactic responses (15, 16). Concurrently, macrophage-derived TNF-α contributes to the neoplastic transformation of gastric epithelial cells via activation of Wnt signaling pathways (17, 18). In the chronic inflammation characterizing HP-related atrophic gastritis, dominance of M1-type macrophages and elevated inducible nitric oxide synthase (iNOS) expression correlate with increased tumorigenic potential, implying that interrupting the M1-to-M2 phenotypic transition may hold therapeutic promise (19, 20). Distinct transcriptional programs drive macrophage polarization: activation of IRF, IFN, and STAT1 favors M1 specification, whereas M2 commitment is promoted via IL-10/STAT3 and IL-4/STAT6 signaling cascades (21). Tumor epithelial cells not only favor *de novo* M2 polarization but can also reprogram existing M1 macrophages toward an M2 phenotype (22, 23). A mechanism underlying this phenotypic conversion involves downregulation of the RNA-binding factor NOVA1, which modulates mRNA stability and intersects with STAT3-mediated signaling (24, 25). The local stroma, particularly subperitoneal fibroblasts and mesenchymal stem cells, plays a key role in skewing macrophage fate toward the M2 subtype (26, 27). Systemic inflammatory mediators further amplify this trend, reinforcing an immunosuppressive environment conducive to metastasis. Recent single-cell RNA-seq studies have revealed transitional macrophage states in gastric tumors, characterized by mixed M1/M2 signatures and enhanced responsiveness to IL-1β and TGF-β cues, suggesting a spectrum rather than a binary phenotype (23, 28, 29). Comparative spatial transcriptomics has further shown that macrophage plasticity is regionally influenced by tumor hypoxia gradients, with perivascular M2-like macrophages expressing high VEGFA and ARG1 levels, contrasting with pro-inflammatory CXCL10^+^ macrophages in the tumor core (30, 31).

### 2.2 Role of TAMs in promoting invasion and metastasis

TAMs promote gastric cancer invasion and metastasis through coordinated regulation of tumor cell motility, extracellular matrix remodeling, vascular remodeling, angiogenesis, lymphangiogenesis, and immune escape (7). TAM-derived exosomes, particularly those enriched in miR-21 and miR-223, suppress PTEN expression and activate the PI3K/AKT pathway, thereby enhancing malignant phenotypes (32, 33). These vesicular signals also induce actin cytoskeletal remodeling and filopodia formation, conferring increased migratory and invasive capacity on recipient tumor cells (34–36). In parallel, TAM-secreted MMP9 activates the PI3K/AKT/Snail cascade, while macrophage-derived TGF-β and IL-6 further reinforce epithelial–mesenchymal transition (EMT) and invasive behavior (37–39). During *Helicobacter pylori* infection, exosomal regulators of epithelial–mesenchymal plasticity can stimulate macrophage-derived IL-1β production, thereby amplifying EMT and metastatic progression (40).

TAMs also coordinate the vascular and lymphatic remodeling required for metastatic dissemination. Within the hypoxic and inflammatory gastric tumor microenvironment, M2-like TAMs interact closely with endothelial cells and release pro-angiogenic mediators (41, 42). Coagulation-related stimuli, including FIII, FVIIa, and FXIIa, induce these macrophages to produce IL-4, IL-10, TGF-β, and TNF-α, which synergistically promote VEGF and MMP9 secretion, endothelial activation, basement membrane degradation, and neovascular expansion (7, 43). TAM-derived Semaphorin 4D further activates EGFR, PKB/AKT, ERK, and STAT3 signaling, thereby contributing to vascular remodeling and tumor neovascularization (44, 45). Alongside angiogenesis, TAMs also regulate lymphatic endothelial function to facilitate lymphangiogenesis, aiding lymphatic infiltration and cancer dissemination (42). Clinically, increased TAM infiltration is closely associated with higher lymphatic vessel density and lymph node metastasis, largely through VEGF-C–mediated signaling (42, 46).

**Figure 1:**
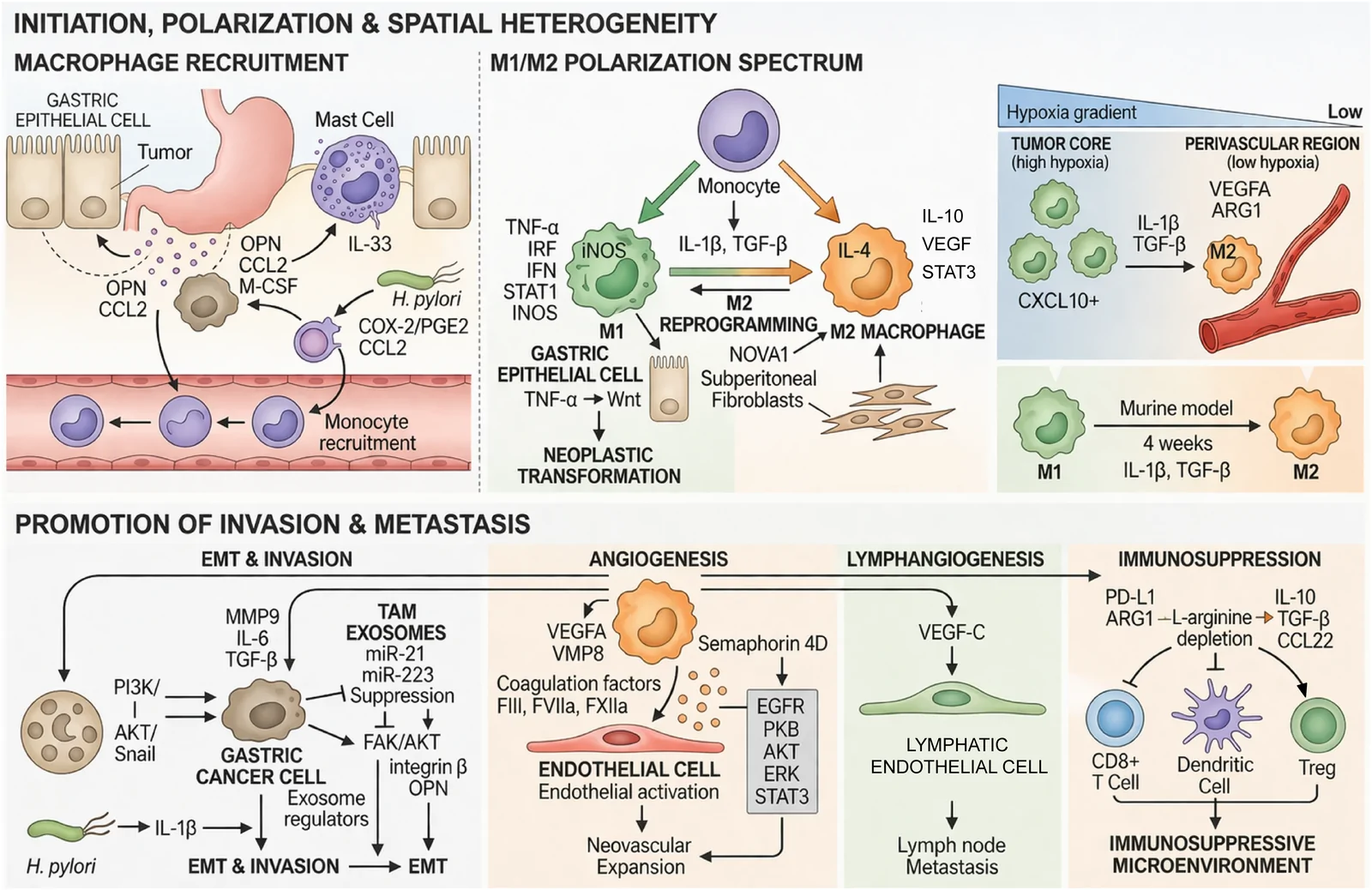
TAM-driven immunosuppressive mechanisms in gastric cancer. The upper panel illustrates the early events governing macrophage recruitment, polarization, and spatial heterogeneity in gastric cancer. At the early stages of GC, tumor-secreted attractants such as osteopontin (OPN) and CCL2 initiate the recruitment of TAMs. CCL2, and M-CSF, together with IL-33 released from mast cells and *Helicobacter pylori*-associated COX-2/PGE2 and CCL2 signaling, promote monocyte recruitment into the tumor microenvironment. Recruited monocytes subsequently differentiate along an M1–M2 polarization spectrum. M1-like macrophages are driven by TNF-α, IRF, IFN, STAT1, and iNOS signaling and exhibit pro-inflammatory properties, whereas M2-like macrophages are induced by IL-4, IL-10/STAT3, ARG1, and VEGF-associated programs and favor tumor progression. Macrophage states remain highly plastic, with reprogramming influenced by IL-1β, TGF-β, fibroblasts, and mesenchymal stromal cells. Spatially, hypoxia gradients further shape macrophage phenotypes, with CXCL10^+^ inflammatory macrophages enriched in the tumor core and M2 macrophages preferentially localized in perivascular regions. The lower panel summarizes the protumor functions of TAMs in gastric cancer progression. TAM-derived cytokines and matrix-remodeling enzymes promote invasion, and metastasis through pathways including PI3K/AKT/Snail, FAK/AKT, and IL-1β-mediated signaling. TAMs also stimulate angiogenesis through VEGFA, MMP9, while VEGF-C secretion promotes lymphangiogenesis and lymph node metastasis. In parallel, TAMs establish an immunosuppressive microenvironment by upregulating PD-L1 and ARG1, depleting L-arginine, and secreting IL-10, TGF-β, and CCL22, thereby suppressing CD8^+^ T cell and dendritic cell function and promoting Treg-mediated immune tolerance. Together, these mechanisms position TAMs as central drivers of gastric cancer progression, metastasis, and resistance to antitumor immunity.

Beyond promoting angiogenesis and invasion, M2-like TAMs exert immunosuppressive effects that hinder T cell–mediated antitumor responses. They secrete IL-10, TGF-β, and CCL22, which inhibit CD8^+^ T cell proliferation, impair dendritic cell function, and recruit Tregs, respectively (47, 48). M2-TAMs also upregulate PD-L1 and express ARG1, leading to L-arginine depletion and impaired T cell receptor signaling (49, 50). This immunosuppressive circuit reduces effector T cell infiltration and cytotoxicity, thereby compromising immune checkpoint inhibitors and adoptive T cell therapies (51). Recent proteomic analyses further revealed that TAM-derived exosomes are enriched in integrin β1 and osteopontin, linking vesicular cargo to FAK/AKT activation and increased peritoneal metastasis in orthotopic models (52–55). Moreover, MMP9 production by M2-TAMs correlates with lymphovascular invasion and poor prognosis, particularly in diffuse-type GC (56, 57) (**Figure 1**).

## 3 Potential mechanisms of TAMs in immunotherapy resistance in gastric cancer

### 3.1 Bridging macrophage polarization and T cell dysfunction in gastric cancer

**Figure 2:**
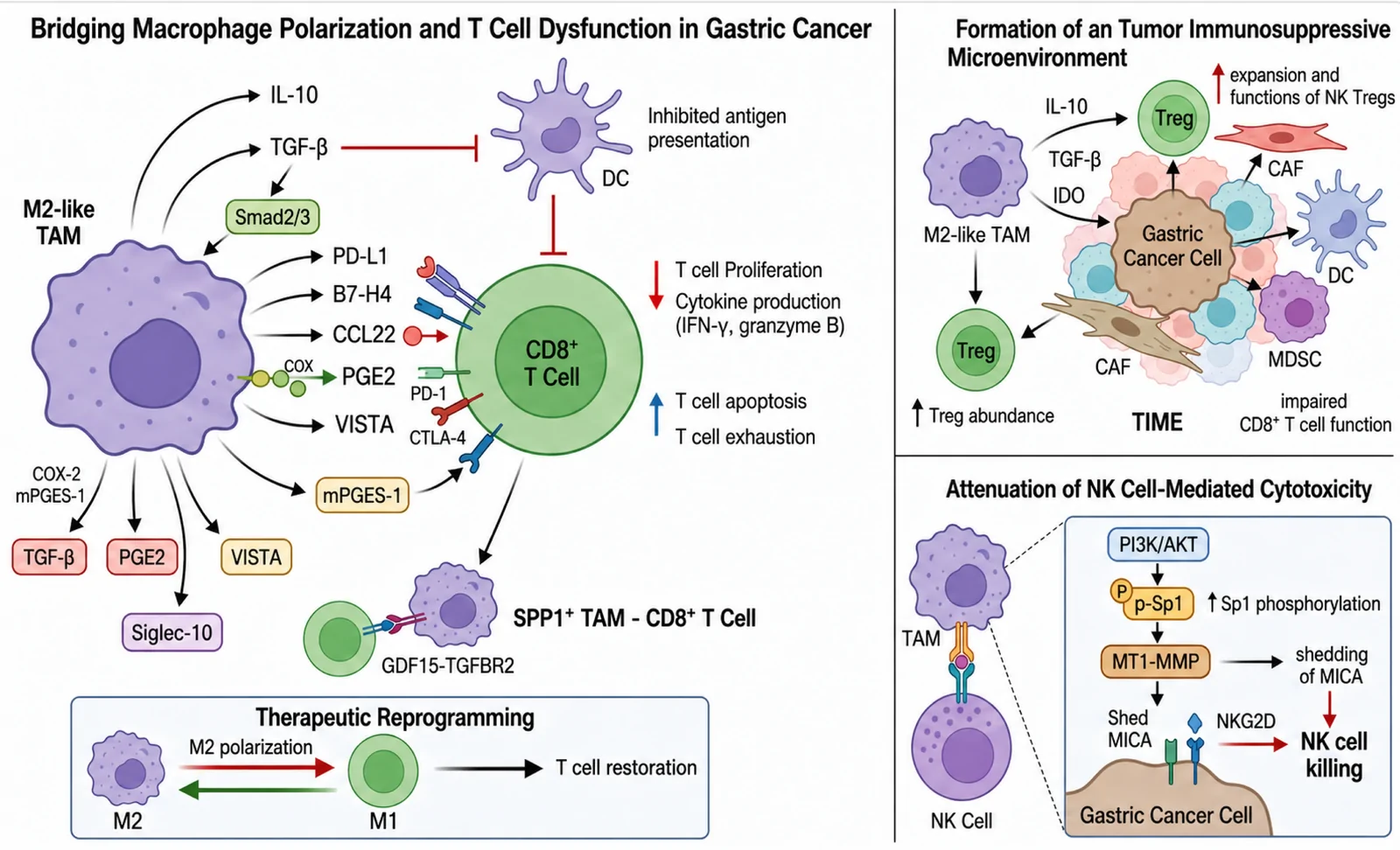
Potential mechanisms of TAMs in immunotherapy resistance in gastric cancer. M2-like TAMs suppress antitumor immunity by linking macrophage polarization to T cell dysfunction, formation of an immunosuppressive tumor immune microenvironment, and impaired NK cell cytotoxicity. TAM-derived IL-10, TGF-β, PGE2, VISTA, PD-L1, B7-H4, CCL22, and Siglec-10 inhibit dendritic cell antigen presentation, promote Treg recruitment, enhance T cell exhaustion, and reduce CD8^+^ T cell proliferation and effector cytokine production. SPP1^+^ TAMs further induce CD8^+^ T cell apoptosis through GDF15–TGFBR2 signaling. In parallel, TAMs cooperate with Tregs, CAFs, DCs, and MDSCs to reinforce immune suppression. The PI3K/AKT–Sp1–MT1-MMP axis promotes MICA shedding, weakening NKG2D-mediated NK cell killing. Reprogramming M2-like TAMs toward M1-like phenotypes may restore antitumor immunity.

TAMs can suppress T-cell activity, impair their ability to recognize and attack tumor cells, thereby enabling tumor cells to evade immune surveillance and ultimately contributing to resistance to immunotherapy. TAMs exert profound effects on adaptive immunity, particularly in shaping the quantity and quality of T cell responses (58). M2-like TAMs secrete high levels of IL-10 and TGF-β, which suppress dendritic cell maturation and inhibit effective antigen presentation, thereby limiting T cell priming and clonal expansion (59, 60). Moreover, M2-TAMs upregulate immune checkpoint ligands such as PD-L1, B7-H4, and VISTA on their surface, promoting T cell exhaustion through engagement of PD-1 and CTLA-4 receptors (61, 62). They also enhance the recruitment of Tregs via CCL22 and inhibit cytotoxic CD8^+^ T cell infiltration by remodeling the extracellular matrix and inducing CXCL12-mediated T cell exclusion (63, 64). TGF-β has been shown to influence the efficacy of PD-1/PD-L1 immunotherapy by suppressing T cell activation and modulating PD-L1 expression. TAM-derived TGF-β inhibits T cell activity by inducing Smad2/3 phosphorylation and suppressing mitochondrial respiration, thereby reducing the expression of IFN-γ and granzyme B in T cells. TGF-β expression is also closely associated with T cell infiltration, and tumors with high TGF-β expression generally exhibit reduced CD8^+^ T cell infiltration (65, 66). In addition, by increasing PD-L1 expression, PGE2 can suppress T-cell activation and function. As a downstream effector of COX-2, PGE2 levels in the tumor microenvironment are regulated by the expression of COX-2 and microsomal PGE synthase-1 (21). Together, these mechanisms contribute to a hostile immune microenvironment that resists T cell-mediated tumor clearance. Therefore, therapeutic strategies that reprogram TAMs toward a pro-inflammatory M1 phenotype or deplete immunosuppressive macrophage subsets may restore T cell functionality and sensitize tumors to subsequent T cell–based immunotherapies (9, 67) (**Figure 2**).

Emerging single-cell and spatial transcriptomic studies further indicate that TAMs in gastric cancer cannot be adequately explained by a simple M1/M2 dichotomy. Instead, distinct macrophage subsets may coexist within the tumor microenvironment and exert divergent effects on immunotherapy response (68, 69). For instance, SPP1^+^ TAMs are frequently associated with metastatic progression, extracellular matrix remodeling, and T-cell dysfunction, whereas CXCL9^+^/CXCL10^+^ inflammatory macrophages may contribute to local immune activation and T-cell recruitment (70, 71). Perivascular VEGFA^+^/ARG1^+^ macrophages appear to support angiogenesis, hypoxia adaptation, and immune exclusion, while VISTA^+^ or Siglec-10-associated macrophage populations may reinforce inhibitory signaling and promote CD8^+^ T-cell exhaustion (41, 72). These findings suggest that macrophage heterogeneity has direct therapeutic implications: depletion of all macrophages may not be optimal, whereas selective targeting or reprogramming of immunosuppressive TAM subsets may better preserve antitumor myeloid functions while overcoming resistance to PD-1/PD-L1 blockade.

### 3.2 Formation of an tumor immunosuppressive microenvironment

The immunosuppressive tumor immune microenvironment in gastric cancer is composed of multiple interacting cellular populations, including cytotoxic T lymphocytes (CTLs), NK cells, cancer-associated fibroblasts (CAFs), myeloid-derived suppressor cells (MDSCs), Tregs, dendritic cells (DCs), and TAMs (73, 74). TAMs secrete immunosuppressive mediators such as IL-10, IDO, and TGF-β, thereby suppress antigen-presenting cell maturation, impair effector T-cell activation, and promote the expansion of suppressive immune cell populations (60). M2-like TAMs promote Treg differentiation through the production of IL-10 and TGF-β, and accelerate Treg recruitment via secretion of CCL22, thereby facilitating the establishment of an immunosuppressive phenotype. In turn, Tregs further stabilize the M2-like macrophage phenotype through immunosuppressive cytokine production, thereby reinforcing a self-sustaining feedback loop of immune suppression (58, 75).

TAMs can secrete a variety of growth factors, cytokines, and proteases that promote tumor growth, invasion, and metastasis while suppressing the activity of immune cells, thereby enabling tumor cells to evade immune surveillance and immune-mediated elimination (58). In addition, TAMs can facilitate immune escape by impairing NK-cell function and reducing their capacity to kill tumor cells (76, 77). *In vivo*, PLEK2 was shown to promote phosphorylation of transcription factor Sp1 through the PI3K/AKT signaling pathway, thereby upregulating the expression of membrane type 1 matrix metalloproteinase (MT1-MMP) (78, 79). This process induced the shedding of human major histocompatibility complex class I chain-related gene A (MICA), preventing NK cells from effectively recognizing and attacking gastric cancer cells and thereby allowing these cells to escape immune destruction (78, 80). Besides, TAMs cooperate with MDSCs and CAFs to further restrict antitumor immunity. TAMs and MDSCs can mutually enhance immunosuppression through arginine metabolism, reactive oxygen species generation, and inflammatory cytokine signaling, collectively weakening CTL and NK-cell cytotoxicity (48, 81, 82). CAFs also interact with TAMs by producing CXCL12, TGF-β, and extracellular matrix components, which limit lymphocyte infiltration and promote immune exclusion (8, 83). Clinically, gastric cancer patients with abundant IL-10-high M2-like TAM infiltration exhibit poorer therapeutic responses and reduced survival after adjuvant chemotherapy, a phenomenon closely associated with increased Treg abundance and impaired CD8^+^ T-cell function (84, 85). Therefore, the presence and activity of TAMs in gastric cancer can impair immune surveillance and tumor clearance, promote tumor progression and metastasis, and represent an important driver of immune evasion in gastric cancer (**Supplementary Table 1**).

### 3.3 TAM-orchestrated stromal and vascular remodeling in immunotherapy resistance

While the direct immunosuppressive effects of TAMs on T cells and NK cells are well-documented, TAMs also confer profound resistance to immunotherapy by actively orchestrating stromal barriers and aberrant vasculature (4, 18, 73). M2-polarized TAMs secrete high levels of TGF-β, IL-10, and PDGF. TAM-derived TGF-β binds to TGF-β receptors (TGFBR2) on resident fibroblasts, triggering the canonical SMAD2/3 signaling pathway (86, 87). This cascade activates the transcription of α-SMA and collagen genes, differentiating normal fibroblasts into CAFs (88, 89). These CAFs subsequently deposit a dense, cross-linked extracellular matrix (ECM) rich in collagen and fibronectin. This desmoplastic stromal barrier acts as a physical shield that not only increases interstitial fluid pressure—limiting drug delivery—but also traps infiltrating CD8^+^ T cells in the peritumoral stroma, preventing their entry into the tumor parenchyma (14, 90–92). Furthermore, CAFs secrete CXCL12, which binds to CXCR4 on T cells, retaining them in the stromal compartment and rendering them susceptible to TAM-derived IL-10-mediated suppression (93, 94). IL-10 signals through the IL-10R/JAK1/STAT3 pathway on DCs, downregulating MHC class II and CD80/CD86, thereby crippling antigen presentation and preventing the priming of new anti-tumor T cell clone (95–98).

Simultaneously, TAMs critically regulate tumor vasculature, another key determinant of immunotherapy resistance. TAMs release VEGF, basic fibroblast growth factor (bFGF), and matrix metalloproteinases (MMPs), which stimulate rapid but chaotic angiogenesis (99, 100). The resulting aberrant vasculature is structurally immature, leaky, and poorly perfused. This abnormal endothelium downregulates adhesion molecules such as ICAM-1 and VCAM-1, and upregulates Fas ligand (FasL), creating an “immune-privileged” vascular barrier that actively prevents the adhesion and extravasation of cytotoxic T lymphocytes (100, 101). Moreover, the leaky and inefficient blood vessels exacerbate intratumoral hypoxia. Hypoxia stabilizes HIF-1α in TAMs, which directly binds to hypoxia-response elements (HREs) in the promoters of target genes (102, 103). This signaling axis not only upregulates the expression of PD-L1 and arginase-1 on TAMs, further suppressing T cell activity, but also induces a positive feedback loop by promoting additional VEGF secretion, thereby perpetuating vascular abnormality and M2 polarization (103, 104). Collectively, the synergistic interplay between TAM-derived TGF-β/IL-10 signaling and VEGF/HIF-1α-mediated vascular remodeling establishes a highly exclusionary, hypoxic, and fibrotic TME. This physical and metabolic fortress fundamentally restricts the infiltration and efficacy of PD-1/PD-L1 blockade, representing a major mechanism of primary and acquired immunotherapy resistance in gastric cancer.

## 4 TAM-based therapeutic strategies against gastric cancer

### 4.1 Therapeutic strategies targeting macrophage polarization

Efforts to therapeutically modulate TAMs in gastric cancer have followed three major strategies: depletion, reprogramming, and recruitment blockade (41). A phase II clinical trial evaluating neoadjuvant docetaxel, cisplatin, and 5-fluorouracil in combination with the PD-L1 inhibitor avelumab for locally advanced gastroesophageal adenocarcinoma reported an expansion of M2-polarized tumor-associated macrophages (M2-TAMs) in non-responding tumors (NCT03288350) (105). CSF1R inhibitors (pexidartinib) reduce TAM density and have shown synergy with checkpoint inhibitors in preclinical models. CD40 agonists, TLR ligands, and HDAC inhibitors can repolarize M2 macrophages toward a pro-inflammatory M1 phenotype, enhancing T cell recruitment and cytotoxicity (106, 107). For example, in murine GC models, activation of CD40 reprogrammed TAMs and restored CD8^+^ T cell infiltration, augmenting PD-1 blockade efficacy (108). CCR2 and CCL2 blockade disrupts monocyte recruitment to tumors, lowering TAM accumulation (109). Intraperitoneal administration of IL-33 induces M2 macrophage polarization by activating the p38–GATA-binding protein 3 signaling pathway and, in synergy with anti-CSF1R therapy, modulates TAMs to suppress gastric cancer metastasis (110).

### 4.2 Chimeric antigen receptor macrophage therapy

CAR-T cell therapy has been extensively investigated in oncology and has shown remarkable efficacy, particularly in hematological malignancies (111, 112). However, its performance in solid tumors remains limited by poor extravasation, insufficient tumor infiltration, functional exhaustion, and the profoundly immunosuppressive TME (113). Several studies have explored the use of genetically engineered monocytes and TAMs as antitumor therapeutic approaches (CAR-M) (114–116). The central principle of this strategy is to endow macrophages with specific antitumor capabilities through genetic engineering, thereby harnessing their inherent biological properties for cancer treatment (117). Klichinsky and colleagues established the feasibility of engineering primary human monocyte-derived macrophages with an anti-HER2 CAR using an Ad5f35 adenoviral platform, demonstrating efficient tumor-directed phagocytosis, M1-like polarization, and significant tumor suppression in xenograft models (118). CAR-M treatment effectively reduced tumor burden and prolonged overall survival compared with control macrophage-treated mice (118). Subsequent studies further showed that anti-HER2 CAR-M not only exerts direct antitumor activity but also enhances the sensitivity of solid tumors to PD-1 blockade by remodeling the local immune microenvironment (119, 120). This encouraging preclinical research has rapidly advanced into phase I clinical trials (NCT06254807 and NCT04660929).

Du et al. developed pArg1-CD47 CAR-Ms by exploiting the intrinsic responsiveness of the macrophage Arg1 promoter, enabling TME-specific activation of cytotoxicity and effectively overcoming SIRPα-mediated inhibition in CD47^+^ cancer cells (121). Other studies have further engineered more sophisticated CAR platforms to disrupt the CD47/SIRPα axis. For example, Chen et al. designed an enhanced synthetic phagocytic receptor (eSPR) that induced sustained M1-like antitumor reprogramming of TAMs, increased direct phagocytosis of tumor cells, and promoted cytotoxic T-cell generation across multiple tumor models (122). Similarly, fusion of a humanized HER2 scFv with the phagocytosis-activating FcγRIIa domain, together with shRNA-mediated SIRPα silencing, conferred CAR-shSIRPα-M with enhanced phagocytic capacity and M1-like antitumor activity (123). In gastric cancer, recent studies have begun to provide direct support for the therapeutic potential of CAR-M. HER2-FcϵR1γ-CAR-engineered peritoneal macrophages specifically targeted HER2-positive gastric cancer cells, promoted tumor regression, and prolonged survival in preclinical models, with further enhancement observed when combined with oxaliplatin (124). In parallel, gastric microbiota-derived signals may further potentiate CAR-M efficacy, as *Ligilactobacillus salivarius*-derived extracellular vesicles activated pro-inflammatory macrophage programs and enhanced the cytotoxic activity of Claudin18.2-targeted CAR macrophages in an FPR1-dependent manner (125). Together, these findings suggest that CAR-M may represent a promising therapeutic strategy for gastric cancer through both direct tumor clearance and macrophage-centered remodeling of the immunosuppressive microenvironment.

## 5 Conclusion

Tumor-associated macrophages (TAMs) have emerged as pivotal regulators of gastric cancer progression, orchestrating tumor growth, metastasis, angiogenesis, immune evasion, and resistance to immune checkpoint inhibitors through complex interactions with tumor cells, stromal components, and other immune populations. Recent advances in single-cell sequencing, spatial transcriptomics, and multi-omics analyses have revealed that TAMs exhibit remarkable spatial and functional heterogeneity that extends far beyond the conventional M1/M2 paradigm. These findings suggest that distinct macrophage subsets may differentially regulate extracellular matrix remodeling, vascular abnormalities, T-cell exclusion, and therapeutic responsiveness. Accordingly, therapeutic strategies are evolving from broad macrophage depletion toward selective macrophage reprogramming and precision targeting. Emerging approaches, including CSF1R and CCR2 blockade, CD40 agonists, and particularly CAR-M therapy, offer promising opportunities to remodel the immunosuppressive tumor microenvironment and enhance the efficacy of existing immunotherapies.

Nevertheless, most macrophage-targeted strategies remain at the preclinical stage, and many of the mechanisms discussed in this review are derived from hypothesis-driven interpretations based on experimental models, requiring validation in well-designed clinical studies. The substantial interpatient variability in TAM composition, plasticity, and spatial organization also presents major challenges for clinical translation, highlighting the need for reliable biomarkers to guide patient selection and monitor therapeutic responses. Furthermore, CAR-M therapy faces several unresolved obstacles, including limited *in vivo* persistence, manufacturing complexity, potential phenotypic instability within the immunosuppressive microenvironment, and the absence of validated macrophage-specific targets for gastric cancer. Future studies integrating spatial multi-omics, longitudinal clinical profiling, and biomarker-guided therapeutic stratification will be essential to define clinically actionable macrophage subsets, optimize combination strategies with immune checkpoint blockade, and facilitate the successful translation of macrophage-directed immunotherapies into precision treatment for gastric cancer.
